# Optimizing hip exoskeleton assistance pattern based on machine learning and simulation algorithms: a personalized approach to metabolic cost reduction

**DOI:** 10.3389/frobt.2025.1669600

**Published:** 2025-09-24

**Authors:** Arash Mohammadzadeh Gonabadi, Iraklis I. Pipinos, Sara A. Myers, Farahnaz Fallahtafti

**Affiliations:** 1 Institute for Rehabilitation Science and Engineering, Madonna Rehabilitation Hospitals, Omaha, NE, United States; 2 Department of Biomechanics, University of Nebraska at Omaha, Omaha, NE, United States; 3 Department of Surgery, University of Nebraska Medical Center, Omaha, NE, United States; 4 Department of Surgery and Research Service, Nebraska-Western Iowa Veterans Affairs Medical Center, Omaha, NE, United States

**Keywords:** hip exoskeleton, machine learning, human-in-the-loop optimization, metabolic cost, personalized wearable robotic control, surrogate modeling, gait optimization, biomechanics

## Abstract

**Introduction:**

Hip exoskeletons can lower the metabolic cost of walking in many tasks and populations, but their assistance patterns must be tailored to each user. We developed a simulation-based, human-in-the-loop (HIL) optimization framework combining machine learning (ML) and global optimization to personalize hip exoskeleton assistance patterns.

**Methods:**

Using data from ten healthy adults, we trained a Gradient Boosting (GB) surrogate model to predict normalized metabolic cost as a function of Peak Magnitude and End Timing of assistive torque. GB achieved the lowest relative absolute error percentage (RAEP) of 0.66%, outperforming Random Forest (RAEP = 0.83%) and Support Vector Regression (RAEP = 0.98%) among nine ML models. We then evaluated seven optimization algorithms, including Covariance Matrix Adaptation Evolution Strategy, Bayesian Optimization, Exploitative Bayesian Optimization, Cross-Entropy, Genetic Algorithm, Gravitational Search Algorithm (GSA), and Particle Swarm Optimization (PSO), to identify optimal assistance profiles.

**Results:**

GSA predicted the lowest metabolic cost (−1.06), equivalent to an estimated 53% reduction relative to no exoskeleton assistance, while PSO showed the highest efficiency (AUC = 0.24).

**Discussion:**

These simulated predictions, though not empirical measurements, demonstrate the framework’s ability to streamline algorithm selection, reduce experimental burden, and accelerate translation of exoskeleton optimization into rehabilitation, occupational, and performance enhancement applications with broader biomechanical and clinical impact.

## 1 Introduction

Mobility disability affects over 26.9% of older adults worldwide (Okoro et al., 2018), contributing to reduced independence, increased healthcare costs, and reduced quality of life (Musich et al., 2018). Wearable robotic assistive devices, such as lower-limb exoskeletons, have emerged as promising tools for improving walking efficiency by reducing the energetic cost of walking (Shepertycky et al., 2021), providing physical support (de Looze et al., 2016; Fox et al., 2019), and restoring weak limb function (Jiryaei et al., 2021). Elevated metabolic cost during walking is a common issue among older adults and individuals with neuromuscular or vascular impairments, often leading to early fatigue, reduced mobility, and a greater risk of sedentary behavior, further exacerbating functional decline and health complications (Das Gupta et al., 2019; Antonellis et al., 2022; Mohammadzadeh Gonabadi et al., 2024c; 2024b). Despite considerable success in laboratory experiments, many exoskeletons have shown limited benefits in real-world applications due to the inherent complexity of human-robot interaction and challenges related to portability and practical deployment outside controlled environments (Hsu et al., 2021; Rodríguez-Fernández et al., 2021; Charette et al., 2023; Mohammadzadeh Gonabadi et al., 2024a). Because the hip can generate substantial positive torque during daily activities, optimally tuned hip exoskeletons have the potential to reduce metabolic cost by up to 40% (Lee et al., 2017; Ding et al., 2018; Seth et al., 2018; Arones et al., 2020; Mohammadzadeh Gonabadi et al., 2020; 2024a). However, personalized tuning is necessary to maximize the benefits of the exoskeleton and human performance, which is challenging outside of a laboratory (Zhang et al., 2017; Slade et al., 2022; Farris et al., 2023).

**FIGURE 1:**
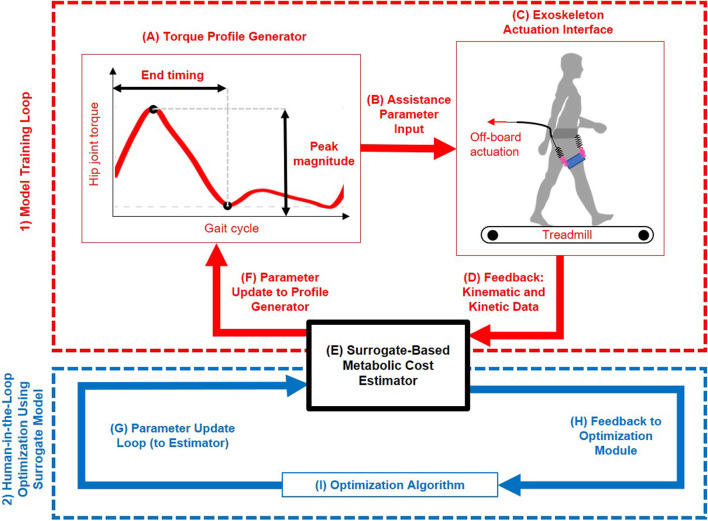
Overview of the human-in-the-loop framework for personalized hip exoskeleton assistance. The system consists of two main loops: (1) Model Training Loop (red): used to generate and collect experimental data (Mohammadzadeh Gonabadi et al., 2024a) for training a surrogate model; and (2) Human-in-the-Loop Optimization Loop Using Surrogate Model (blue): used to optimize assistance parameters based on surrogate-predicted metabolic cost iteratively. **(A)** Torque Profile Generator defines a piecewise semi-linear hip torque profile based on Peak Magnitude and End Timing. **(B)** Assistance Parameter Input transmits these parameters to the exoskeleton controller. **(C)** Exoskeleton Actuation Interface applies the assistive torque to the user during treadmill walking via off-board actuation. **(D)** Feedback: Kinematic and Kinetic Data are collected through motion capture and force plate instrumentation. **(E)** Using a trained machine learning model, a Surrogate-Based Metabolic Cost Estimator predicts metabolic cost based on biomechanical input data. **(F)** Parameter Update to Profile Generator enables exploration of new assistance conditions during model training. **(G)** Parameter Update Loop (to Estimator) delivers proposed assistance parameters from the optimizer to the surrogate model for evaluation. **(H)** Feedback to Optimization Module returns the surrogate-predicted metabolic cost associated with each evaluated parameter set. **(I)** Optimization Algorithm iteratively selects parameter sets that minimize predicted metabolic cost. This framework enables efficient, data-driven tuning of hip exoskeleton assistance, reducing reliance on repeated experimental measurements while supporting individualized optimization.

Human-in-the-loop (HIL) optimization has been developed as a method for real-time personalization of exoskeleton parameters. The structure of this HIL optimization strategy is shown in the upper (red) loop of Figure 1, where human feedback is used to adjust assistance parameters systematically. In this process, device control is iteratively adjusted to enhance user performance based on physiological feedback during real-time use (Ding et al., 2018; Rayssiguie and Erden, 2022; Farris et al., 2023; Lakmazaheri et al., 2024; Firouzi et al., 2025; Lin et al., 2025). HIL optimization has substantially improved exoskeleton performance across various activities, including recent translation into real-world conditions (Koller et al., 2016; Malcolm et al., 2017; Sreenivasa et al., 2017; Zhang et al., 2017). However, assessing key performance metrics such as metabolic rate often involves costly equipment and prolonged steady-state walking, making these experiments challenging (Mohammadzadeh Gonabadi et al., 2020; Mohammadzadeh Gonabadi et al., 2024a; Antonellis et al., 2022). To get around these challenges, simulation methods using surrogate models have become more common. These models approximate the relationship between assistance parameters and physiological outcomes using Machine Learning (ML) techniques trained on experimental data (Kutulakos and Slade, 2024). Once validated, surrogate models enable rapid and cost-effective testing of numerous assistance parameter combinations within the simulation framework, eliminating the need for additional human trials. The lower (blue) loop in Figure 1 outlines this surrogate-based optimization framework, where ML predictions guide iterative parameter refinement without requiring direct physiological measurements. Recent studies used data-driven methods to estimate within-stride metabolic cost during walking (Gonabadi et al., 2020; Antonellis et al., 2022; Dzewaltowski et al., 2024; Mohammadzadeh Gonabadi et al., 2024b; Mohammadzadeh Gonabadi et al., 2024c). They showed that variations in biomechanical variables can predict energy expenditure with high temporal resolution and greater consistency compared to earlier model-based approaches (Gonabadi et al., 2020; Antonellis et al., 2022; Dzewaltowski et al., 2024; Mohammadzadeh Gonabadi et al., 2024b; Mohammadzadeh Gonabadi et al., 2024c). Although these models cannot fully capture the complexities of human adaptation, they offer meaningful insights into parameter sensitivity and can guide experimental design.

Complementing data-driven methods, biomechanics-based optimization approaches have leveraged musculoskeletal modeling and control theory to design torque profiles that minimize joint loads and enhance gait stability (Desplenter and Trejos, 2018; Baud et al., 2021; Firouzi et al., 2025). Research on human-adaptation dynamics has revealed how motor co-adaptation and neuromuscular adjustments shape user responses to exoskeleton assistance, underscoring the importance of adaptive strategies (Poggensee and Collins, 2021; Echeveste and Bhounsule, 2025). Energy efficiency modeling in wearables has also progressed through predictive frameworks that account for device mass, actuation efficiency, and user biomechanics to estimate potential metabolic savings (Slade et al., 2022; Chang et al., 2023; Scherb et al., 2023; Dzewaltowski et al., 2024). Despite these advances, prior HIL optimization approaches often require lengthy experimental sessions that induce fatigue, limit scalability, and underrepresent inter-individual biomechanical variability (Slade et al., 2022; Dzewaltowski et al., 2024). Our simulation-based framework addresses these shortcomings by employing surrogate models informed by population-level data to benchmark global optimization algorithms, reduce experimental demands, and enable preliminary strategy identification for real-world translation in rehabilitation and occupational contexts.

Recent research highlights the effectiveness of ML-based approaches in various exoskeleton applications. Kutulakos and Slade (Kutulakos and Slade, 2024) modeled metabolic landscapes for ankle exoskeletons using Gaussian Process regression and simulated HIL optimization under various user and device scenarios. Their findings confirmed that surrogate-based frameworks can replicate trends observed in real-time optimization and inform decisions regarding algorithm selection and parameter tuning. Gonabadi et al. (Mohammadzadeh Gonabadi et al., 2024b) demonstrated the feasibility of using artificial neural networks to estimate metabolic cost directly from ground reaction forces and joint moments, offering a real-time alternative to indirect calorimetry. Their findings highlight the predictive strength of biomechanical inputs and support the integration of ML in assistive device development and gait analysis applications. In another study (Mohammadzadeh Gonabadi et al., 2024c), nonlinear dynamical measures and artificial intelligence algorithms were used to classify gait patterns, illustrating the capability of AI in capturing subtle variations in neuromotor control. These results indicate that ML models can be widely applied to personalize gait optimization (Mohammadzadeh Gonabadi et al., 2024c). Similar techniques have been tested on hip–knee–ankle exoskeletons and soft wearable systems (Peng et al., 2020), confirming the broad usefulness of this approach. Complementing these, advanced fractional-order optimization techniques, such as tempered fractional gradient descent (Naifar, 2025) and gradient-based algorithms for conformable fractional derivatives (Alaia et al., 2025), provide robust frameworks for handling non-integer order dynamics in learning applications. Such approaches could enhance surrogate model training and optimal control in exoskeleton systems by improving convergence in noisy or fractional biomechanical landscapes. While not the present study’s focus, these methods highlight valuable avenues for future extensions of surrogate-based HIL optimization.

The present study introduces a surrogate-based optimization framework for hip exoskeleton assistance (Mohammadzadeh Gonabadi et al., 2024a). We developed a simulation-based framework to predict and optimize the metabolic cost of hip exoskeleton assistance. Using experimental data, we trained predictive models and integrated them into a computational pipeline to identify effective assistance strategies without additional human trials. This study evaluated the accuracy of surrogate models and compared optimization algorithm performance in identifying low metabolic cost hip exoskeleton assistance parameters. We introduced a simulation-based approach for hip exoskeletons that uses a highly accurate model to enable fast and effective personalization. We hypothesized that BO would outperform heuristic methods like PSO due to its probabilistic acquisition strategy, which balances global exploration with focused exploitation, an essential advantage in metabolic optimization tasks with limited evaluation budgets.

## 2 Methods

### 2.1 Experimental data

This study utilized a previously published dataset collected during treadmill walking with a bilateral semi-rigid hip exoskeleton designed to assist hip extension through torque application during early stance (Mohammadzadeh Gonabadi et al., 2024a). Ten healthy adults with no known disabilities (4 males, six females; age: 27.6 ± 5.9 years; body mass: 65.3 ± 13.1 kg; height: 1.66 ± 0.08 m) completed a single experimental session consisting of walking trials under multiple assistance conditions. Participants walked at a constant speed of 1.25 m/s on an instrumented split-belt treadmill (Bertec, Columbus, OH, United States). Three-dimensional lower-limb kinematics were captured using a 10-camera motion capture system operating at 120 Hz (Vicon, Oxford, United Kingdom). In comparison, ground reaction forces were recorded at 1000 Hz using embedded force plates (Bertec, Columbus, OH, United States). Marker trajectories and force data were time-synchronized and filtered using a low-pass fourth-order Butterworth filter (6 Hz for kinematics, 20 Hz for kinetics). The hip exoskeleton (Mohammadzadeh Gonabadi et al., 2024a) was powered by an off-board rotary motor system (HuMoTech, Pittsburgh, PA, United States) that delivered bilateral torques through a series-elastic actuator coupled to the user’s thighs via custom-fitted cuffs. A real-time controller (Gonabadi et al., 2020; Antonellis et al., 2022; Mohammadzadeh Gonabadi et al., 2024a) was implemented using a Simulink model executed on a SpeedGoat real-time target machine (SpeedGoat, Bern, Switzerland), interfaced with the hardware through a custom control box (Gonabadi et al., 2020; Antonellis et al., 2022; Mohammadzadeh Gonabadi et al., 2024a). The assistive torque followed a piecewise semi-linear profile with three key parameters: torque onset (fixed at 90% of the gait cycle, just before heel strike), Peak Magnitude (ranging from 0.04 to 0.14 Nm/kg), and End Timing (ranging from 21% to 49% of the gait cycle) (Mohammadzadeh Gonabadi et al., 2024a). Peak torque was always delivered at 17% of the gait cycle (early stance), while variations in End Timing modulated the duration of assistance (Mohammadzadeh Gonabadi et al., 2024a).

Twelve walking conditions were tested, consisting of ten powered assistance profiles, one PowerOff condition (with the exoskeleton worn but not actuated), and one NoExo condition (without the device) (Mohammadzadeh Gonabadi et al., 2024a). Only the ten powered assistance conditions were used for surrogate model development and simulation-based HIL optimization. These conditions represented active device use and captured a range of assistive torque profiles relevant for optimization. Each condition was performed for a sufficient duration (at least 6 min) to ensure steady-state metabolic measurement using indirect calorimetry. Net metabolic cost (W/kg) was computed by subtracting standing baseline values and was normalized to the PowerOff condition to derive a percentage change in metabolic cost per trial. Across all conditions, normalized net metabolic cost ranged from −34.91% to +49.76%. A negative value indicates a reduction in metabolic cost relative to PowerOff, whereas a positive value reflects an increase. We chose the PowerOff condition as the reference point, as was done in the original study. This approach allowed for a balanced comparison that included metabolic reductions and increases, enabling consistent modeling of the cost landscape across assistance parameters (Mohammadzadeh Gonabadi et al., 2024a). A combination of Peak Magnitude and End Timing values characterized each assistance condition. These parameters were extracted for modeling purposes and used as inputs for surrogate model training. A few missing parameter values (e.g., for rare outliers or corrupted sensor readings) were imputed using condition-type means based on Peak Magnitude category (e.g., low, medium, high). This ensured a complete dataset for subsequent ML model development.

This study utilized an existing dataset collected from 10 healthy adults during a previously approved experimental protocol (Mohammadzadeh Gonabadi et al., 2024a) at the University of Nebraska Medical Center Institutional Review Board (protocol number: 0101-19-FB; initial approval: 22 April 2019). All participants provided written informed consent before participation. The present work involved only secondary analysis and modeling of this dataset; no new human experiments were conducted.

### 2.2 Simulated methods

Experimental walking data from a previous study involving ten healthy adults were used to train multiple ML models to predict normalized metabolic cost (Mohammadzadeh Gonabadi et al., 2024a). These included Gradient Boosting (GB), a powerful ensemble method that builds models sequentially to minimize prediction error using stage-wise additive modeling and decision trees (Friedman, 2001). GB builds a predictive model by combining many simple decision trees, much like a team of experts refining predictions by learning from each other’s mistakes. Support Vector Regression (SVR) was employed for its ability to model high-dimensional nonlinear relationships using kernel functions (Smola and Schölkopf, 2004). Polynomial Ridge (PR) and Linear Ridge (LR) regressions provided interpretable linear models with L2 regularization to mitigate overfitting and multicollinearity (Hoerl and Kennard, 2000; Hastie et al., 2009). Random Forest (RF), another ensemble method, aggregates predictions from multiple decision trees to capture nonlinear interactions and reduce variance in the model output (Breiman, 2001). We also included four variants of Gaussian Process (GP) regression (Rasmussen, 2004), providing probabilistic predictions and quantifying uncertainty in model estimates. The Gaussian Process Absolute Exponential (GPAE) kernel is suitable for modeling moderately smooth functions (Rasmussen, 2004). In contrast, the Gaussian Process Matern 3/2 (GPM) kernel offers a flexible balance between model smoothness and responsiveness to local variations in data (Rasmussen, 2004). The Gaussian Process Rational Quadratic (GPRQ) kernel captures data patterns with multiple length scales and is often used when the smoothness of the underlying function is not known *a priori* (Rasmussen, 2004). Finally, the Gaussian Process Squared Exponential (GPSE) kernel, also known as the radial basis function, assumes highly smooth underlying functions. It is widely used in surrogate modeling due to its strong generalization ability (Rasmussen, 2004). This diverse set of regressors enabled robust modeling of the complex relationship between assistance parameters and metabolic cost across multiple function classes and regularization strategies.

Although metabolic landscapes were simulated, they were directly trained on experimental data from 10 healthy adults. The surrogate model was therefore not arbitrary but grounded in real measurements, serving as a computational approximation of user feedback. This framework enabled controlled benchmarking of optimization algorithms while avoiding the burden of repeated long-duration experiments. Importantly, in real-world HIL optimization, algorithms would operate with live user feedback, whereas the surrogate provides a reproducible and safe environment for preliminary evaluation.

Although individual metabolic landscapes are known to vary due to physiological and neuromuscular differences, this study pooled data across 10 healthy subjects to train a population-level surrogate model. The intention was not to create a fully personalized model for each subject but to capture generalizable trends in the relationship between assistance parameters and metabolic cost. This surrogate served as a representative user feedback model, enabling simulation-based evaluation of various optimization algorithms in a controlled environment. The study’s main goal was to determine which optimization strategy is most effective for human-in-the-loop exoskeleton parameter tuning. In future real-time applications, the selected algorithm will be applied directly to honest user feedback to personalize assistance. At the same time, the surrogate model will remain a simulation tool for preliminary exploration.

The chosen models cover a spectrum of learning approaches, including linear (LR, PR), non-parametric (GP), and ensemble-based (GB, RF), to thoroughly explore the metabolic cost profile. These surrogates were integrated with seven global optimization algorithms to evaluate their ability to identify low-cost assistance profiles. Covariance Matrix Adaptation Evolution Strategy (CMAES) is a generative, model-free, and sample-efficient local optimizer that adapts its search distribution based on the best-performing parameter sets in each generation, making it effective for noisy and non-convex problems (Hansen and Ostermeier, 2001). Bayesian Optimization (BO) uses a probabilistic surrogate, often a Gaussian process, and an acquisition function to balance exploration and exploitation, enabling sample-efficient global optimization in high-dimensional parameter spaces (Snoek et al., 2012). Exploitative Bayesian Optimization (EBO) is a variant of BO with a lower exploration constant, biasing the search toward local exploitation, which has been shown to enhance convergence in scenarios with low measurement noise or time-varying optima (Kutulakos and Slade, 2024). The Cross-Entropy (CE) method is a generative optimization technique that updates a probability model using top-performing samples. It encourages exploration but usually needs more evaluations than BO or CMAES (Deng, 2006). The Genetic Algorithm (GA) applies biologically inspired operations such as selection, crossover, and mutation to evolve solutions across generations. It is valued for its straightforward implementation and strong global search performance (Holland, 1992; Gonabadi, 2016; Mohammadzadeh Gonabadi et al., 2017a). Inspired by Newtonian gravity, the Gravitational Search Algorithm (GSA) models the agents as objects attracted to each other based on their masses. This provides a flexible and innovative approach to solving multidimensional optimization problems (Rashedi et al., 2009). Lastly, Particle Swarm Optimization (PSO) models the collective behavior of swarms and uses social and cognitive components to improve candidate solutions iteratively. Its rapid convergence and simplicity have made it a popular choice for nonlinear optimization problems (Coello et al., 2004; Mohammadzadeh et al., 2011; Mohammadzadeh Gonabadi et al., 2017b). PSO mimics a flock of birds searching for food, where each particle adjusts its path based on its own and the group’s best positions. A summary of all ML models and optimization algorithms is provided in Table 1.

**TABLE 1:** Machine learning models and global optimization algorithms**.** The listed models were used to construct surrogate predictors of metabolic cost, while the optimization algorithms were applied to identify low-cost assistance parameters based on the surrogate models.

#	Machine learning models	Optimization algorithms
1	Linear Ridge (LR)	Covariance Matrix Adaptation Evolution Strategy (CMAES)
2	Polynomial Ridge (PR)	Bayesian Optimization (BO)
3	Support Vector Regression (SVR)	Exploitative Bayesian Optimization (EBO)
4	Random Forest (RF)	Cross-Entropy Method (CE)
5	Gradient Boosting (GB)	Genetic Algorithm (GA)
6	Gaussian Process – Absolute Exponential (GPAE)	Gravitational Search Algorithm (GSA)
7	Gaussian Process – Matern 3/2 (GPM)	Particle Swarm Optimization (PSO)
8	Gaussian Process – Rational Quadratic (GPRQ)	
9	Gaussian Process – Squared Exponential (GPSE)	

### 2.3 Data augmentation

A simplified biomechanical representation of hip assistance was created, modeling the exoskeleton torque profile as a piecewise semi-linear function (Kutulakos and Slade, 2024; Mohammadzadeh Gonabadi et al., 2024a). The profile increased semi-linearly from zero to Peak Magnitude until the defined End Timing, then returned to zero by the end of the gait cycle. This approximation reflected the actual assistive torque behavior described in the experimental conditions (Kutulakos and Slade, 2024; Mohammadzadeh Gonabadi et al., 2024a). To improve generalizability and enable robust model training, the original experimental dataset was augmented using synthetically generated trials (Kutulakos and Slade, 2024). Each data point was perturbed with zero-mean Gaussian noise (standard deviation = 0.05), corresponding to approximately 4.6% of the parameter range. This approach, previously employed in simulation studies of metabolic landscapes for exoskeleton optimization (Kutulakos and Slade, 2024), replicates experimental variability and enriches the surrogate model’s learning capacity (Kutulakos and Slade, 2024). Gaussian noise augmentation has improved the accuracy and robustness of surrogate models, particularly in regression tasks with limited data availability (Kutulakos and Slade, 2024). The synthetic dataset and the original powered trials were used as input for training ML models to predict normalized metabolic cost (Kutulakos and Slade, 2024). These predictions then served as the foundation for surrogate-based optimization simulations.

To improve model robustness, 20 augmented samples were generated for each of the 100 experimental trials by adding zero-mean Gaussian noise (σ = 0.05, ∼4.6% of the normalized range) to the input parameters (Peak Magnitude and End Timing). This produced 2000 synthetic trials in total. Unlike prior work that perturbed metabolic cost to simulate measurement noise (Kutulakos and Slade, 2024), we perturbed inputs to approximate variability in how assistance parameters may vary across repeated walking bouts. This broadened the surrogate’s training distribution while preserving physiologically plausible ranges.

For each experimental trial, inspired by (Kutulakos and Slade, 2024), 20 synthetic samples were generated by perturbing the assistance parameters—Peak Magnitude and End Timings—using zero-mean Gaussian noise with a standard deviation of 0.05, equivalent to ∼4.6% of the normalized parameter range [0, 1]. These perturbations were applied solely to the input parameters, while the corresponding normalized metabolic cost was retained unchanged to preserve label fidelity (Kutulakos and Slade, 2024). This augmentation approach aimed to simulate inter-trial variability observed in experimental protocols and enhance the robustness and generalization of surrogate model training (Kutulakos and Slade, 2024).

### 2.4 Data preparation and normalization

The combined dataset consisted of 100 valid experimental trials and 200 synthetic trials, totaling 300 samples. This augmentation enhanced the surrogate model’s generalization ability across diverse assistance profiles (Kutulakos and Slade, 2024). Synthetic trials were generated to simulate physiological variability, ensuring robust training for machine learning models (Kutulakos and Slade, 2024). Input features included Peak Magnitude and End Timing, and the target variable was normalized metabolic cost. All variables were normalized to the range [0, 1] using min-max scaling to standardize the feature space and improve model convergence. For each feature x , the normalized value $x_{\text{norm}}$ was computed as shown in Equation 1:

$$x_{\text{norm}}=\frac{x-x_{\min}}{x_{\max}-x_{\min}}$$ (1)

where $x_{\min}$ and $x_{\max}$ are the minimum and maximum values of the feature across the dataset.

### 2.5 Machine learning (ML) model: training and hyperparameters

Additional polynomial terms (e.g., squared features) were included for PR to capture potential nonlinear interactions. The resulting feature matrix and target vector were used for training and evaluating all surrogate models (Kutulakos and Slade, 2024). Nine regression models were developed and evaluated: LR, PR, SVR, RF, GB, and four variants of GP models—GPAE, GPM, GPRQ, and GPSE. Each model was trained using five-fold cross-validation (k = 5) repeated across 100 iterations, yielding 500 (5 × 100) randomized splits (Kutulakos and Slade, 2024; Mohammadzadeh Gonabadi et al., 2024b; Mohammadzadeh Gonabadi et al., 2024c; Gonabadi et al., 2025). For each iteration, K-Fold cross-validation (k = 5) was applied, with 80% of the data (n = 240 trials) used for training and 20% (n = 60) for testing in each split (Kutulakos and Slade, 2024; Mohammadzadeh Gonabadi et al., 2024b; Mohammadzadeh Gonabadi et al., 2024c; Gonabadi et al., 2025).

Model-specific configurations included the use of L2 regularization (λ = 1) for both LR and PR (Hoerl and Kennard, 2000; Hastie et al., 2009), with PR incorporating a second-degree polynomial expansion to account for nonlinear trends (Kutulakos and Slade, 2024). SVR was trained using standardized inputs to enable nonlinear regression mapping based on kernel transformations (Kutulakos and Slade, 2024). RF was implemented with 100 trees using the bagging method to reduce variance and mitigate overfitting (Kutulakos and Slade, 2024). GB used the LSBoost method with 100 learning cycles to iteratively minimize prediction error (Kutulakos and Slade, 2024). All GP models (GPAE, GPM, GPRQ, GPSE) were implemented using exact fitting and prediction methods (Rasmussen, 2004; Kutulakos and Slade, 2024), each employing a different kernel function to reflect varying assumptions about the smoothness and structure of the input space (Rasmussen, 2004; Kutulakos and Slade, 2024). These diverse model architectures were chosen to reflect a range of learning biases, from linear parametric models to non-parametric probabilistic approaches, supporting robust surrogate construction based on the selected assistance parameters (Kutulakos and Slade, 2024).

Similar to the literature (Slade et al., 2022; Kutulakos and Slade, 2024; Echeveste and Bhounsule, 2025), hyperparameters for all models were tuned using a grid search within 5-fold cross-validation. For GB, we optimized learning rate (0.01–0.1), maximum tree depth (3–7), and number of estimators (100–500). Gaussian Process kernels were selected based on empirical fit (e.g., SE, Matern, Rational Quadratic). Ridge regressions used L2 regularization strength determined via cross-validation. These procedures minimized RAEP and ensured consistent performance across folds.

Recent advances in deep stable learning for handling imbalanced datasets (Xu et al., 2025) provide additional methodological support for our surrogate modeling strategy. While their application focused on fault diagnosis in engineering systems, the underlying principles of improving robustness and predictive reliability are directly transferable to biomechanical optimization, where data heterogeneity and limited sample sizes often present similar challenges.

To prevent overfitting, we employed repeated 5-fold cross-validation (500 iterations), regularization where available, and constrained model complexity (e.g., limiting GB tree depth). These measures improved generalizability and reduced the risk of models capturing noise rather than meaningful trends (Slade et al., 2022; Kutulakos and Slade, 2024; Echeveste and Bhounsule, 2025).

### 2.6 Model evaluation

Model performance was evaluated using Relative Absolute Error (RAE) and Relative Absolute Error Percentage (RAEP) across the 100 × 5-fold cross-validation scheme. For each test sample i , the relative absolute error $\text{RA}E_{i}$ and RAEP(%) were calculated as shown in Equations 2, 3:

$$\text{RA}E_{i}=\frac{\left|y_{\text{actual},i}-y_{\text{pred},i}\right|}{y_{\text{actual},i}}$$ (2)

$$\text{RAEP}\left(\%\right)=\left(\frac{1}{n}\sum_{i=1}^{n}\text{RA}E_{i}\right)\times 100$$ (3)

where $y_{\text{actual},i}$ and $y_{\text{pred},i}$ are the ground-truth and predicted normalized metabolic cost for the $i_{\text{th}}$ trial, respectively, and n is the number of test samples per split. RAEP reflects the average percentage deviation from actual values, normalized to ground truth, and allows for consistent model comparison regardless of cost magnitude. The distributions of RAEP across all 500 splits were aggregated and visualized to assess each model’s accuracy and robustness. Models with lower RAEP and more concentrated error distributions were considered superior in terms of generalization and suitability for use in surrogate-based optimization.

### 2.7 Optimization and hyperparameters

The most accurate surrogate model will simulate the metabolic landscape with the highest accuracy and guide parameter optimization. Seven global optimization algorithms—CMAES, BO, EBO, CE, GA, GSA, and PSO—were implemented to identify assistance parameters (Peak Magnitude and End Timing) that minimized predicted metabolic cost. Each optimization algorithm was executed across 10 independent trials (re-running 10 times) to ensure robustness against stochastic variability in initialization and search trajectory, with 200 evaluations per trial (a total of 10 × 200 evaluations) to reflect a realistic limit on experimental feasibility in HIL studies (Kutulakos and Slade, 2024; Mohammadzadeh Gonabadi et al., 2024b; Mohammadzadeh Gonabadi et al., 2024c; Gonabadi et al., 2025). Gaussian noise (standard deviation = 0.046) was added to the surrogate model outputs to simulate inter-trial variability and measurement noise typically observed in metabolic cost estimation, improving ecological validity of the optimization simulation (Kutulakos and Slade, 2024). Assistance parameters were bounded within the normalized range [0, 1].

CMAES was initialized with a population size of 15, a mean vector of (0.5, 0.5), and a step-size (σ) of 0.3, using an elite size of 3 to guide distribution adaptation (Hansen and Ostermeier, 2001). BO employed an expected-improvement-plus acquisition function with an exploration constant of 2.6 (Snoek et al., 2012), while EBO used the same framework but with a reduced exploration constant of 0.93 to favor local exploitation (Kutulakos and Slade, 2024). CE operated with a population size of 15, an elite fraction of 0.5 (Deng, 2006), and a similar initialization as CMAES (Hansen and Ostermeier, 2001). GA used a population 20 with an 80% crossover rate (Holland, 1992; Gonabadi, 2016; Mohammadzadeh Gonabadi et al., 2017a). GSA configured 20 agents with a gravitational constant of 100 and an acceleration constant (α) of 20 (Rashedi et al., 2009). Finally, PSO simulated 20 particles with inertia-based position updates for global search (Coello et al., 2004; Mohammadzadeh et al., 2011; Mohammadzadeh Gonabadi et al., 2017b). These configurations were chosen to represent a spectrum of exploration-exploitation strategies and computational complexities, allowing for a comprehensive assessment of algorithmic suitability for surrogate-based optimization in exoskeleton applications (Kutulakos and Slade, 2024).

Similar to sustainability-driven optimization in other engineering domains, such as additive manufacturing (Oladunni et al., 2025), our framework emphasizes computational efficiency to minimize resource demands and enhance scalability. While their application focused on reducing greenhouse gas emissions, the methodological parallels underscore how optimization strategies can be leveraged to improve exoskeleton control systems and streamline personalization.

To ensure comparability across optimization algorithms, all methods were initialized using consistent strategies, supported by setting a fixed random seed (rng (42)) for reproducibility. CMAES and CE began with a mean vector of (0.5, 0.5) and a standard deviation (σ) of 0.3. BO and EBO used normalized input bounds with fixed exploration constants of 2.6 and 0.93, respectively. GA, GSA, and PSO initialized their populations (or particles/agents) uniformly across the normalized parameter space [0, 1]^2^ using identical seeds. These design choices reduced initialization bias, ensuring that differences in performance arose from algorithm dynamics rather than initial sampling variance. All optimization parameters and configurations are summarized in Table 2 and detailed further in the supplementary code repository.

**TABLE 2:** Summary of optimization algorithm configurations and initialization settings. All algorithms operated within normalized bounds [0, 1] for both Peak Magnitude and End Timings. The maximum number of function evaluations was set to 200 per run for all methods.

Algorithm	Population Size/Agents	Initialization Mean/Method	Key parameters	Random seed used
CMA-ES	15	Mean: (0.5, 0.5) σ = 0.3	λ = 15 Elite size = 3	rng (42)
Bayesian Optimization (BO)	N/A (sequential)	Uniform sampling in [0, 1]^2^	Acquisition: EI+ Exploration constant = 2.6	rng (42)
Exploitative BO (EBO)	N/A (sequential)	Uniform sampling in [0, 1]^2^	Acquisition: EI+ Exploration constant = 0.93	rng (42)
Cross-Entropy (CE)	15	Mean: (0.5, 0.5) σ = 0.3	Elite fraction = 0.5 λ = 30 (2× CMAES)	rng (42)
Genetic Algorithm (GA)	20	Uniform sampling in [0, 1]^2^	Crossover fraction = 0.8 Generations = ceil (200/20)	rng (42)
Gravitational Search Algorithm (GSA)	20	Uniform sampling in [0, 1]^2^	G_0_ = 100; α = 20 Max iterations = ceil (200/20)	rng (42)
Particle Swarm Optimization (PSO)	20	Uniform sampling in [0, 1]^2^	Inertia-based updates Swarm size = 20; Iterations = ceil (200/20)	rng (42)

### 2.8 Performance metrics

Optimization algorithm performance was evaluated using two complementary metrics: Area Under the Curve (AUC) and Average Rate of Improvement (ARI) (Bartz-Beielstein et al., 2010; Kuyu and Vatansever, 2019; Franks et al., 2021; Tyagi et al., 2024). AUC measures the overall optimization efficiency by quantifying the area between the evolving minimum predicted metabolic cost and the known global minimum (Bartz-Beielstein et al., 2010; Kuyu and Vatansever, 2019; Franks et al., 2021; Tyagi et al., 2024). Specifically, AUC captures how closely the best-found cost during each iteration approaches the global minimum over all 200 evaluations, normalized for interpretability (Bartz-Beielstein et al., 2010; Kuyu and Vatansever, 2019; Franks et al., 2021; Tyagi et al., 2024). A lower AUC indicates that the algorithm consistently identifies cost-effective solutions throughout the optimization trajectory (Bartz-Beielstein et al., 2010; Kuyu and Vatansever, 2019; Franks et al., 2021; Tyagi et al., 2024). Mathematically, AUC was computed using Equation 4:

$$\text{AUC}=\frac{1}{N}\sum_{i=1}^{N}\left|{\hat{y}}_{\min}^{\left(i\right)}-y_{\min}\right|$$ (4)

where ${\hat{y}}_{\min}^{\left(i\right)}$ is the minimum predicted metabolic cost at the *i*th iteration, $y_{\min}$ is the global minimum cost (−1.12), and $\left(N=200\right)$ is the total number of evaluations for each optimization algorithm trial.

On the other hand, ARI quantifies the average per-iteration improvement in cost by computing the mean absolute reduction in the best-found value between consecutive evaluations (Bartz-Beielstein et al., 2010; Kuyu and Vatansever, 2019; Franks et al., 2021; Tyagi et al., 2024). Higher ARI values denote faster convergence toward optimal solutions (Bartz-Beielstein et al., 2010; Kuyu and Vatansever, 2019; Franks et al., 2021; Tyagi et al., 2024), which is particularly valuable in HIL scenarios where experimental trials are limited. ARI was calculated using Equation 5:

$$\text{ARI}=\frac{1}{N-1}\sum_{i=2}^{N}\left|{\hat{y}}_{\min}^{\left(i\right)}-{\hat{y}}_{\min}^{\left(i-1\right)}\right|$$ (5)

In general, N represents the number of evaluations in a given optimization run, which can vary if an optimizer terminates early. In this study, however, we fixed $\left(N=200\right)$ for all algorithms to maintain identical evaluation budgets and enable fair comparisons across optimizers. These metrics—adapted from best practices in benchmarking optimization algorithms (Bartz-Beielstein et al., 2010; Kuyu and Vatansever, 2019; Franks et al., 2021; Tyagi et al., 2024)—provide a comprehensive view of both optimization efficiency (AUC) and convergence dynamics (ARI) (Bartz-Beielstein et al., 2010; Kuyu and Vatansever, 2019; Franks et al., 2021; Tyagi et al., 2024). ARI shows the progression speed of each algorithm. ARI is defined as the mean absolute change in minimum cost per iteration, normalized by the total number of evaluations. This metric captures the dynamic improvement profile of the optimizer across its search trajectory, providing a measure of how quickly the algorithm approaches optimality. This is particularly valuable in human-in-the-loop applications where time efficiency and early gains are often more desirable than only final performance, especially in clinical populations with limited tolerance for prolonged testing (Bartz-Beielstein et al., 2010; Kuyu and Vatansever, 2019; Franks et al., 2021; Tyagi et al., 2024).

ARI and AUC are particularly suited for assessing algorithm suitability as exoskeleton parameter tuning, where convergence speed and consistency affect user comfort, time efficiency, and clinical feasibility (Bartz-Beielstein et al., 2010; Kuyu and Vatansever, 2019; Franks et al., 2021; Tyagi et al., 2024).

## 3 Results

### 3.1 Model performance

**FIGURE 2:**
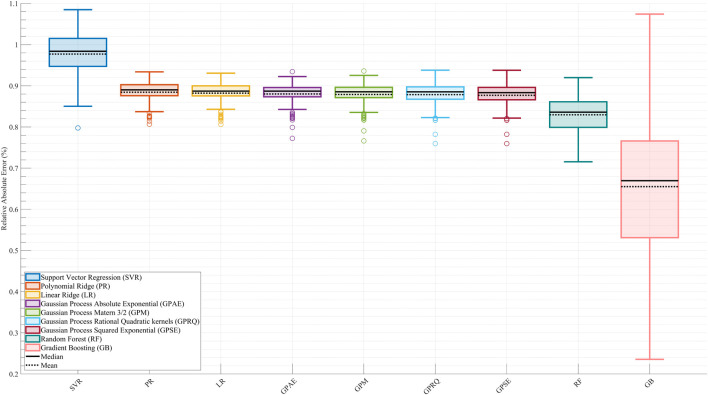
Comparison of surrogate model performance based on Relative Absolute Error Percentage (RAEP). Boxplots show RAEP distributions across all cross-validation splits for nine machine learning models: Linear Ridge (LR), Polynomial Ridge (PR), four Gaussian Process (GP) variants—Squared Exponential (GPSE), Matern 3/2 (GPM), Absolute Exponential (GPAE), and Rational Quadratic (GPRQ)—Support Vector Regression (SVR), Random Forest (RF), and Gradient Boosting (GB). GB achieved the lowest RAEP (0.66%) with greater variability, indicating high accuracy with some sensitivity to data distribution. SVR had the highest error (0.98%), while GP models and linear methods showed similar intermediate performance (0.88%). RF (0.83%) performed better than most but did not surpass GB. The solid black line in each boxplot represents the median RAEP value, and the dashed black line denotes the mean. While RAEP indicates that all models achieve relatively low average errors across cross-validation splits, this metric reflects generalizability on test sets and does not capture trial-specific deviations or nonlinear dynamics.

Figure 2 displays the RAEP distributions for each of the nine surrogate models across all cross-validation splits. GB achieved the lowest RAEP (0.66%) among all models, indicating the highest predictive accuracy. This model also exhibited greater variability in distribution, likely due to its responsiveness to localized error reduction. RF followed with an RAEP of 0.83%, outperforming all linear models and kernel-based methods. In contrast, SVR showed the poorest performance with an RAEP of 0.98%, reflecting challenges in capturing the nonlinearities and variations of the metabolic cost surface. All four GP models—GPSE, GPM, GPAE, and GPRQ—and the LR and PR models produced nearly identical RAEP values of 0.88%. These results suggest that while GP models provided smooth and stable estimates, they did not outperform tree-based ensembles in this dataset. LR and PR also failed to achieve an acceptable accuracy, likely due to their limited ability to model variations and nonlinear interactions in the metabolic cost time profile. The boxplots reveal that while GB delivered the lowest median and mean RAEP, it also had a broader interquartile range, reflecting higher sensitivity to data distribution and outliers. This trade-off suggests that GB offers the most flexible and accurate surrogate for capturing complex cost patterns, at the expense of higher variability in certain conditions.

**FIGURE 3:**
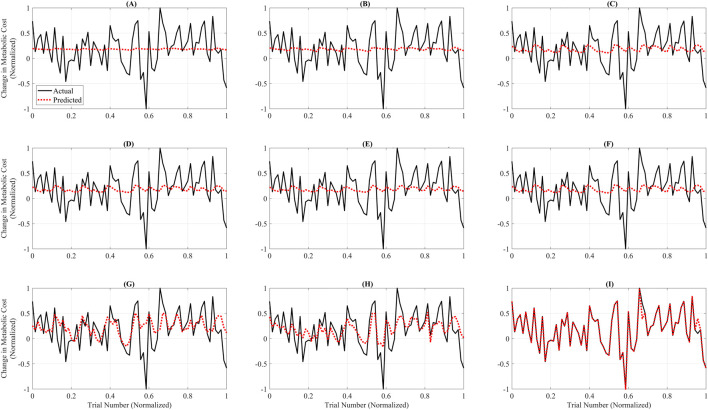
Trial-wise comparison of actual versus predicted metabolic cost across all models. Each subplot shows predicted (red dashed line) and actual (black line) normalized metabolic cost across 100 test trials for a single model. The x-axis represents trial indices normalized to the [0, 1] range to emphasize pattern alignment and model comparison rather than raw trial numbering. The subplots correspond to: **(A)** Linear Ridge (LR), **(B)** Polynomial Ridge (PR), **(C)** Gaussian Process Squared Exponential (GPSE), **(D)** Gaussian Process Matern 3/2 (GPM), **(E)** Gaussian Process Absolute Exponential (GPAE), **(F)** Gaussian Process Rational Quadratic (GPRQ), **(G)** Support Vector Regression (SVR), **(H)** Random Forest (RF), and **(I)** Gradient Boosting (GB). GB **(I)** exhibited the closest agreement with actual values, accurately tracking the trend and magnitude. RF **(H)** and GP models **(C–F)** showed moderate fidelity, whereas LR **(A)**, PR **(B)**, and SVR **(G)** failed to capture rapid changes or peak variations. These plots emphasize GB’s superior predictive performance and trial-level reliability for metabolic cost estimation. Unlike Figure 2, which summarizes average errors, this figure emphasizes trial-specific fidelity using models trained on the full dataset. Linear and Polynomial Ridge produce flatter predictions and fail to capture trial-level nonlinearities, whereas Gradient Boosting aligns more closely with actual data, underscoring its superior expressiveness.

Figure 3 illustrates the trial-wise comparison of actual versus predicted normalized metabolic cost across the complete dataset for each surrogate model. Among all models, GB (Figure 3I) demonstrated the highest alignment between predicted and actual trends, closely tracking the full dynamic range of trial-level metabolic responses. The model captured peaks and valleys with minimal lag, reflecting its strong temporal fidelity and low overall RAEP. RF (Figure 3H) and the GP models—GPSE, GPM, GPAE, and GPRQ (Figures 3C–F)—showed moderate agreement with actual values but exhibited some smoothing, particularly in capturing rapid fluctuations. While they followed general trends, their predictions tended to underestimate trial-to-trial variability. Linear models LR and PR (Figures 3A,B) showed the weakest performance, failing to capture significant inflection points and producing flat or lagged predictions. SVR (Figure 3G) struggled particularly in low-cost regions, displaying more frequent divergence from the ground truth. These differences highlight the limitations of models that lack the expressive capacity needed to model nonlinear trial-level dynamics. Figure 3 reinforces the conclusion that GB offers the best overall predictive accuracy and provides the most reliable trial-level performance across varying metabolic cost profiles.

**FIGURE 4:**
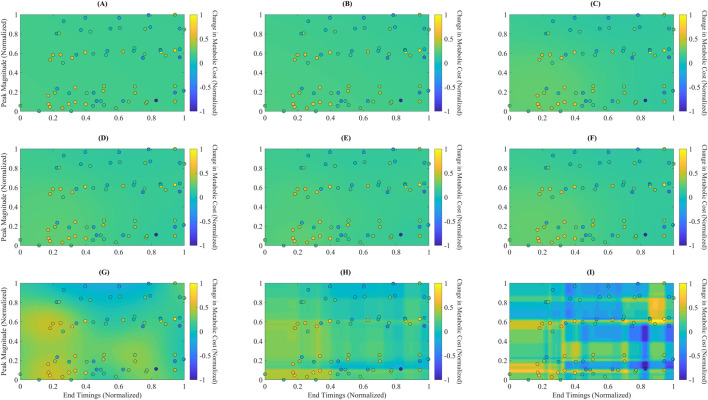
Predicted metabolic landscapes with overlaid actual data points for each surrogate model. Filled contour plots represent predicted normalized metabolic cost over the assistance parameter space, defined by End Timing (x-axis) and Peak Magnitude (y-axis). Overlaid circles indicate the actual experimental data points used for training. The subplots correspond to: **(A)** Linear Ridge (LR), **(B)** Polynomial Ridge (PR), **(C)** Gaussian Process Squared Exponential (GPSE), **(D)** Gaussian Process Matern 3/2 (GPM), **(E)** Gaussian Process Absolute Exponential (GPAE), **(F)** Gaussian Process Rational Quadratic (GPRQ), **(G)** Support Vector Regression (SVR), **(H)** Random Forest (RF), and **(I)** Gradient Boosting (GB). GB **(I)** generated the most detailed and responsive cost surface, capturing sharp gradients and nonlinear parameter interactions. RF **(H)** and GP models **(C–F)** showed smoother interpolations with moderate spatial resolution. SVR **(G)** produced more diffuse gradients, while LR **(A)** and PR **(B)** failed to reflect meaningful topographic variation. These visualizations support GB’s superior spatial expressiveness and consistency with the actual data distribution.

Although LR and PR achieved relatively low RAEP values (∼0.88%) in the cross-validation analysis (Figure 2), their performance was not considered acceptable for surrogate-based optimization. This is because optimization requires models that capture localized trial-to-trial variations and nonlinear dynamics of the metabolic cost landscape. Figures 3, 4 show that LR and PR produced flatter predictions and featureless landscapes, failing to represent critical nonlinearities between assistance parameters and metabolic cost. The apparent discrepancy between Figures 2, 3 arises from their distinct methodologies: Figure 2 reports averaged RAEP from cross-validation, which masks trial-specific deviations, whereas Figure 3 evaluates trial-level fidelity using models trained on the full dataset (Slade et al., 2022; Kutulakos and Slade, 2024; Echeveste and Bhounsule, 2025). Thus, while RAEP confirms that LR and PR can achieve low average error, their inability to reproduce nonlinear dynamics makes them less reliable compared to GB and RF, which better capture complex cost patterns.

Figure 4 presents each surrogate model’s predicted metabolic time profiles using filled contour plots, with overlaid actual data points shown as circles. The plots depict each model’s mapping of the normalized assistance parameter space, characterized by Peak Magnitude (y-axis) and End Timing (x-axis), to the corresponding predicted changes in normalized metabolic cost. Notably, GB (Figure 4I) captures sharp transitions and localized cost gradients, showing a highly responsive and detailed cost surface. The filled contours in the GB plot reveal strong differentiation across regions, consistent with the model’s ability to generalize complex, nonlinear interactions between parameters. In contrast, LR (Figure 4A) and PR (Figure 4B) produce relatively flat and featureless landscapes, suggesting limited ability to represent nonlinear cost changes. GP models—GPSE, GPM, GPAE, and GPRQ (Figures 4C–F)—generate smoothly varying surfaces that interpolate well across the space but lack high-frequency response near dense data clusters. This reflects their kernel-driven behavior, which favors continuity over local sensitivity. SVR (Figure 4G) shows a more spread-out prediction pattern with rough gradients, failing to align closely with the actual metabolic cost pattern. RF (Figure 4H) demonstrates improved spatial granularity, particularly near more concentrated regions of the parameter space, though its boundaries remain blocky due to the discrete nature of tree-based predictions. These landscape visualizations reinforce earlier quantitative findings: GB provides the most spatially expressive and data-consistent model, making it the most appropriate choice for surrogate-based optimization of exoskeleton assistance.

### 3.2 Optimization results

Optimization results are summarized in Table 3. Compared to all other algorithms, GSA reached the lowest normalized metabolic cost (−1.06), indicating the most considerable reduction over the PowerOff baseline. This solution was located at (Peak Magnitude = 0.20) and (End Timing = 0.83), a region identified across multiple high-performing methods. PSO also reached near-optimal cost (−1.00), but with a much lower Mean ARI (0.30 × 10^−5^), indicating slower convergence despite strong endpoint performance. BO and EBO predicted final costs of −0.999 and −0.990, respectively, with moderate AUC values (BO: 0.32, EBO: 0.27), highlighting efficient early search performance. EBO reached the highest Mean ARI (3.29 × 10^−5^), suggesting the most rapid cost reduction per iteration. In contrast, CMAES and CE resulted in higher costs (−0.68 and −0.74, respectively) and less efficient convergence (AUC: 0.56 and 0.46). GA produced the highest final cost (0.40), exceeding the baseline, and confirming the weakest optimization performance in this context.

**TABLE 3:** Optimization results summary**.** Summary of performance metrics for the seven optimization algorithms integrated with the Gradient Boosting (GB) surrogate model. The table includes: (1) the number of evaluations to convergence, (2) the mean computational time in seconds, (3) the optimal assistance parameters—Peak Magnitude and End Timing—identified by each algorithm, (4) the mean normalized metabolic cost achieved at convergence, (5) the mean area under the convergence curve (AUC) as a measure of optimization efficiency, and (6) the average rate of improvement (ARI) representing convergence speed. Algorithms include Covariance Matrix Adaptation Evolution Strategy (CMAES), Bayesian Optimization (BO), Exploitative Bayesian Optimization (EBO), Cross-Entropy (CE), Genetic Algorithm (GA), Gravitational Search Algorithm (GSA), and Particle Swarm Optimization (PSO). To improve clarity for biomechanics-focused readers, we provide brief interpretations of the key metrics used in this study. The Relative Absolute Error Percentage (RAEP) quantifies how closely the surrogate model predicts metabolic cost relative to experimental ground truth, with lower RAEP reflecting greater predictive accuracy and alignment with physiological outcomes. The Area Under the Convergence Curve (AUC) measures the efficiency of an optimizer by integrating error reduction across iterations; a smaller AUC indicates faster and more reliable convergence toward an optimal assistance profile. The Adjusted Rank Index (ARI) evaluates the consistency and robustness of optimizer performance across repeated simulations, helping to identify stable strategies that are more likely to generalize in practice. The predicted metabolic cost provides surrogate-based estimates of energy expenditure under different assistance profiles, normalized to baseline walking, where lower values suggest potential reductions in user effort. Finally, optimization runtime represents computational latency in simulation rather than biological stabilization time, allowing algorithms to be compared on efficiency while acknowledging that human-in-the-loop experiments are dominated by physiological adaptation timescales.

Algorithm	Number of evaluations	Mean time (s)	Peak magnitude	End timing	Normalized Metabolic cost	Mean AUC	Mean ARI
CMAES	200.00	0.60	0.44	0.68	−0.68	0.56	1.85 × 10^−5^
BO	200.00	26.01	0.12	0.80	−1.00	0.32	2.32 × 10^−5^
EBO	200.00	24.01	0.19	0.82	−0.99	0.27	3.29 × 10^−5^
CE	200.00	0.60	0.42	0.79	−0.74	0.46	2.58 × 10^−5^
GA	200.00	0.73	0.10	0.56	0.40	0.33	2.57 × 10^−5^
GSA	200.00	0.59	0.20	0.83	−1.06	0.61	2.21 × 10^−5^
PSO	200.00	1.26	0.13	0.82	−1.00	0.24	0.30 × 10^−5^

**FIGURE 5:**
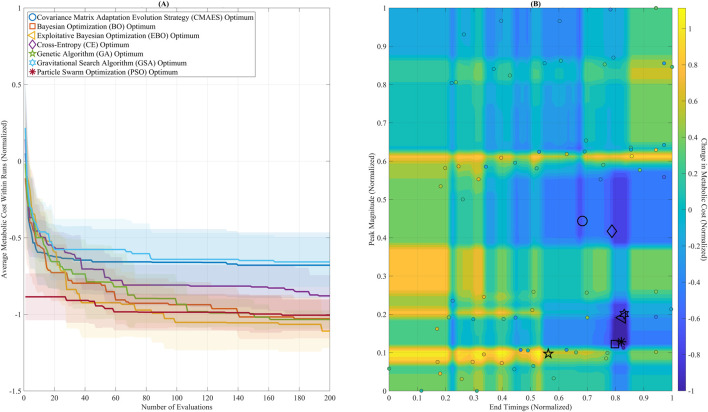
Optimization convergence and parameter landscape. **(A)** Convergence trajectories of seven global optimization algorithms over 200 evaluations, using the Gradient Boosting (GB) surrogate model. Algorithms include: Covariance Matrix Adaptation Evolution Strategy (CMAES), Bayesian Optimization (BO), Exploitative Bayesian Optimization (EBO), Cross-Entropy (CE), Genetic Algorithm (GA), Gravitational Search Algorithm (GSA), and Particle Swarm Optimization (PSO). BO, EBO, and PSO reached near-optimal solutions within the first 100 evaluations. GSA ultimately reached the lowest final cost but showed greater variability across iterations. **(B)** The GB-predicted metabolic cost surface is visualized as a filled contour plot across the assistance parameter space (Peak Magnitude and End Timing). Final optimal solutions from each algorithm are plotted, showing that most solutions converged within a low-cost region between Peak Magnitude 0.10–0.20 and End Timing 0.75–0.85.

Figure 5 shows optimization outcomes using the GB model’s convergence trajectories (Figure 5A) and the predicted metabolic pattern (Figure 5B). In Figure 5A, BO, EBO, and PSO converged rapidly within the first 100 evaluations, with EBO showing the most rapid decline, aligning with its high ARI. While ultimately reaching the lowest cost, GSA showed greater variance over time, consistent with its higher AUC (0.61). Figure 5B displays the optimal solutions from all algorithms on the GB-predicted cost surface. Most optima are clustered within a region defined by Peak Magnitude = (0.10–0.20) and End Timing = (0.75–0.85), representing a biomechanically meaningful and metabolically favorable range. This agreement across algorithms validates the surrogate model’s reliability in guiding the search toward physiologically optimal assistance profiles.

**FIGURE 6:**
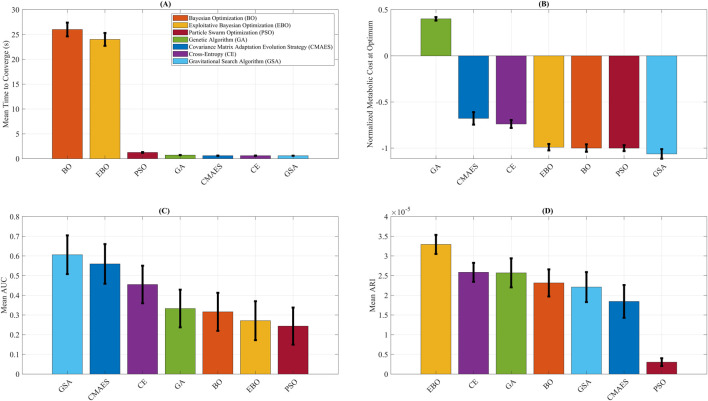
Optimization algorithm performance comparison. Summary of key performance metrics for Covariance Matrix Adaptation Evolution Strategy (CMAES), Bayesian Optimization (BO), Exploitative Bayesian Optimization (EBO), Cross-Entropy (CE), Genetic Algorithm (GA), Gravitational Search Algorithm (GSA), and Particle Swarm Optimization (PSO). **(A)** Mean time to convergence, highlighting the higher computational cost of BO and EBO relative to other methods. **(B)** Final normalized metabolic cost predicted by each algorithm, with GSA attaining the lowest value. **(C)** Mean area under the convergence curve (AUC), where lower values indicate higher optimization efficiency. **(D)** Average rate of improvement (ARI), representing the steepness of per-iteration cost reduction, with EBO yielding the highest ARI. The error bars reflect variability across repeated optimization runs, providing uncertainty quantification for convergence efficiency and predicted metabolic cost outcomes. To improve clarity for biomechanics-focused readers, we provide brief interpretations of the key metrics used in this study. The Relative Absolute Error Percentage (RAEP) quantifies how closely the surrogate model predicts metabolic cost relative to experimental ground truth, with lower RAEP reflecting greater predictive accuracy and alignment with physiological outcomes. The Area Under the Convergence Curve (AUC) measures the efficiency of an optimizer by integrating error reduction across iterations; a smaller AUC indicates faster and more reliable convergence toward an optimal assistance profile. The Adjusted Rank Index (ARI) evaluates the consistency and robustness of optimizer performance across repeated simulations, helping to identify stable strategies that are more likely to generalize in practice. The predicted metabolic cost provides surrogate-based estimates of energy expenditure under different assistance profiles, normalized to baseline walking, where lower values suggest potential reductions in user effort. Finally, optimization runtime represents computational latency in simulation rather than biological stabilization time, allowing algorithms to be compared on efficiency while acknowledging that human-in-the-loop experiments are dominated by physiological adaptation timescales.

Figure 6 summarizes the performance of all optimization algorithms using bar plots for key evaluation metrics. Figure 6A shows the average convergence time. BO and EBO required the longest mean time to converge (26.01 s and 24.01 s, respectively), reflecting the added computational cost of their model-fitting steps. In contrast, CMAES, CE, GA, and GSA ALL converged in less than 1 s, while PSO required a moderate 1.26 s to converge. These runtimes represent computational latency only and do not account for biological stabilization times inherent to human experiments. Figure 6B presents the normalized metabolic cost at optimum. Among all algorithms, GB predicted the largest metabolic reduction, with an optimal normalized metabolic cost of −1.06, corresponding to a 53% predicted reduction compared to the no-assistance baseline. It should be noted that this reduction reflects model-based predictions rather than direct human metabolic measurements. PSO, BO, and EBO converged to similarly low values near −1.00, indicating strong final performance. CE and CMAES reached higher-cost plateaus (−0.74 and −0.68, respectively), while GA performed poorly with a final cost of 0.40, failing to reduce metabolic cost. Figure 6C displays the mean AUC, a measure of optimization efficiency. PSO predicted the lowest AUC (0.24), followed by EBO (0.27) and BO (0.32), indicating compelling early exploration and convergence. In contrast, GSA had the highest AUC (0.61), suggesting slower but ultimately effective convergence. Figure 6D compares the ARI across algorithms. EBO predicted the highest Mean ARI (3.29 × 10^−5^), reflecting the steepest per-iteration improvement. CE (2.58 × 10^−5^) and GA (2.57 × 10^−5^) also demonstrated strong ARI values, while BO and GSA showed moderate improvement rates (2.32 × 10^−5^ and 2.21 × 10^−5^, respectively). Despite its substantial final cost, PSO had the lowest ARI (0.30 × 10^−5^), indicating slower per-step progress.

## 4 Discussion

This study assessed the predictive accuracy of surrogate models and compared the performance of optimization algorithms in identifying metabolically efficient hip exoskeleton assistance settings. It was hypothesized that BO, with its probabilistic acquisition mechanism that strategically balances exploration and exploitation, would surpass heuristic approaches such as PSO, particularly in scenarios constrained by limited evaluation resources. Our findings partially supported this hypothesis. By achieving a 53% metabolic cost reduction within seconds, the proposed simulation-based surrogate modeling and optimization framework significantly reduces experimental burden and supports real-time adaptive control, advancing the deployment of personalized exoskeleton strategies. While BO and EBO demonstrated high convergence efficiency and strong predictive accuracy when coupled with the GB surrogate model, PSO and GSA also showed exceptional performance. Specifically, GSA predicted the lowest predicted normalized metabolic cost (−1.06), and PSO recorded the most efficient convergence (AUC = 0.24). These outcomes suggest that, although BO-based strategies are effective in data-limited conditions, heuristic methods such as PSO and GSA remain highly competitive when paired with robust surrogate models.

### 4.1 Modeling the metabolic landscape

Consistent with prior efforts to simulate HIL optimization using surrogate models for ankle exoskeletons (Kutulakos and Slade, 2024), this study constructed metabolic cost landscapes for hip exoskeleton assistance using a broad array of ML models. Kutulakos and Slade (Kutulakos and Slade, 2024) used several variants of GP regression to generate synthetic metabolic landscapes and reported an average prediction error of approximately 10% on their ankle exoskeleton dataset. They emphasized that this error level is relatively low given the estimated 5% standard deviation of first-order metabolic cost measurements (Zhang et al., 2017). In comparison, our best-performing model, GB, achieved a markedly lower RAEP of 0.66%, substantially improving upon the 10% benchmark and indicating greater predictive fidelity in capturing the relationship between assistance parameters and metabolic cost in the context of hip exoskeletons. While the four GP variants used in our study—GPSE, GPM, GPAE, and GPRQ—produced stable RAEP values (∼0.88%), consistent with the performance range reported in (Kutulakos and Slade, 2024), they were outperformed by ensemble-based models. GB showed higher sensitivity to localized variations and better captured inter-subject metabolic trends, while RF (RAEP = 0.83%) also demonstrated solid performance. These results suggest that tree-based models may better capture complex, non-linear patterns commonly seen in hip assistance settings. Moreover, unlike the surrogate training in (Kutulakos and Slade, 2024), which relied on unaugmented data, we expanded our dataset by adding Gaussian noise (mean = 0, SD = 0.05) to simulate physiological and sensor variability, thereby increasing model robustness. This augmentation enabled the ML models to reflect real-world uncertainties in metabolic measurement, ultimately improving their reliability for downstream optimization tasks. In addition, Monteiro et al. (Monteiro et al., 2024) implemented an EGPR model to estimate real-time metabolic cost during HIL optimization for knee exoskeleton assistance. They reported an RAEP of 26% across five participants, which is still higher than the error observed in our study (Monteiro et al., 2024). Altogether, these findings highlight the effectiveness of GB for modeling the metabolic landscape in HIL simulation, providing accurate predictions compared to traditional GP models and greater robustness for practical optimization. GB’s sequential error correction and ability to capture localized nonlinear patterns explain its superior performance over GP models, which assume smoother cost landscapes.

Although RAEP is a standard metric for evaluating surrogate model accuracy, in this study, it yielded uniformly low values across all models, making it less informative for distinguishing performance differences among optimizers. For this reason, RAEP should be interpreted cautiously and primarily as a measure of overall model fit, while metrics such as AUC and ARI provide more meaningful insight into optimizer efficiency and convergence behavior.

While the surrogate landscape provides a computationally efficient means to test optimization strategies, it remains an approximation of real physiology. As such, the reported metabolic reductions represent model-based predictions rather than empirical measurements. Future validation in live HIL trials is required to confirm the translational relevance of these findings. Uncertainty was evaluated using multiple complementary approaches. Gaussian Process models provided predictive confidence intervals, while ensemble methods (GB, RF) allowed assessment of stability via tree variance. Additionally, variability across 500 cross-validation splits highlighted sensitivity to training/test partitioning. Together, these measures provided insight into model robustness and the reliability of surrogate predictions (Slade et al., 2022; Kutulakos and Slade, 2024; Echeveste and Bhounsule, 2025).

### 4.2 Simulating HIL optimization

This study advances simulation-based HIL optimization by evaluating multiple global algorithms for identifying individualized hip exoskeleton assistance parameters. The optimization framework yielded a metabolic cost reduction of up to 53%, with GSA achieving the lowest normalized metabolic cost (−1.06) at a Peak Magnitude of 0.20 and End Timing of 0.83. This assistance timing aligns with late stance, coinciding with peak activity of the hip extensors, and is supported by prior biomechanical studies emphasizing late-phase torque application to reduce muscular demand (Mohammadzadeh Gonabadi et al., 2020; Rodríguez-Fernández et al., 2021). While BO and EBO were expected to outperform due to their probabilistic acquisition strategies that balance global exploration with exploitation (Kutulakos and Slade, 2024), PSO showed superior efficiency (AUC = 0.24) with a modest computation time of 1.26 s, leveraging swarm intelligence to rapidly converge on high-performing parameter sets (Lee et al., 2017). EBO predicted the steepest convergence (ARI = 3.29 × 10^−5^), followed by BO (ARI = 2.32 × 10^−5^), though both required longer runtimes (24–26 s). CMAES, CE, GSA, and GA all converged in less than 1 s, but CMAES and CE lagged in cost minimization, and GA failed to reach a meaningful minimum. Gaussian noise (σ = 0.046) and 10 repeated trials of 200 evaluations ensured robust convergence and reproducibility (Kutulakos and Slade, 2024).

Our findings partially diverge from Kutulakos and Slade (Kutulakos and Slade, 2024), who simulated HIL optimization for ankle exoskeletons and found that BO and EBO converged fastest (∼60 evaluations) in a 4-parameter space (peak time, rise time, fall time, and peak torque), outperforming CMAES and CE. While their CMAES showed robustness in high-dimensional and time-varying settings, our results showed CMAES trailing behind BO-based methods in a simpler 2D space. This likely reflects BO’s higher exploration constant (Snoek et al., 2012), facilitating broader sampling and more efficient convergence in low-dimensional landscapes (Kutulakos and Slade, 2024). Comparisons with experimental HIL benchmarks underscore the practical implications of these findings. Zhang et al. reported a 17% ± 3% metabolic reduction with CMAES over ∼2 h for ankle exoskeleton optimization (Zhang et al., 2017); Ding et al. predicted a 17.4% ± 3.2% reduction with BO for a hip exosuit in ∼90 min (Ding et al., 2018); and Slade et al. predicted a 23% ± 8% reduction using short walking bouts with BO for ankle exoskeleton in a real-world setting (Slade et al., 2022). Our surrogate-based framework predicted a 53% reduction in seconds, reinforcing its time-efficiency and optimization efficacy for pre-tuning and algorithm evaluation before live deployment.

The broad low-cost region identified by the surrogate landscape (Peak Magnitude = 0.10–0.20, End Timing = 0.75–0.85; Figure 5B) suggests that diverse parameter sets may yield similar benefits, offering greater flexibility for adaptive controllers than the narrow optima often observed in experimental HIL studies (Zhang et al., 2017; Ding et al., 2018; Slade et al., 2022; Wang et al., 2022). Moreover, the optimal End Timing of 0.83 supports phase-aligned torque delivery targeting peak hip extensor activity (Mohammadzadeh Gonabadi et al., 2020), in contrast to ankle-focused strategies that support plantarflexors during push-off (Slade et al., 2022). The moderate Peak Magnitude of 0.20 Nm/kg mitigates over-assistance risks while maintaining biomechanical synergy (Lin et al., 2025). The reliability of PSO and BO/EBO supports their potential integration into adaptive control systems. At the same time, the fast execution times of CMAES and CE may suit applications requiring low-latency updates. Practically, this framework enables metabolic cost reduction in clinical rehabilitation (e.g., improving endurance for individuals with mobility impairments), enhances safety in occupational applications (e.g., reducing fatigue during heavy labor), and boosts athletic training efficiency. However, the relatively longer runtimes of BO and EBO (∼25 s) indicate that faster algorithms like CMAES or CE may be more appropriate for real-time adaptation scenarios.

To ground our parameterization biomechanically, the two torque parameters studied—Peak Magnitude and End Timing—were selected because they represent the most influential biomechanical factors in hip exoskeleton assistance. Peak Magnitude defines the level of external torque applied to the hip extensors during stance, directly influencing muscle activation and joint loading, while End Timing determines when assistance is withdrawn, shaping the transition into swing and neuromuscular adaptation. Prior experimental studies have shown that both magnitude and timing critically modulate metabolic cost and gait stability, supporting their selection as physiologically meaningful variables (Gonabadi et al., 2020; Antonellis et al., 2022; Dzewaltowski et al., 2024; Mohammadzadeh Gonabadi et al., 2024a; Mohammadzadeh Gonabadi and Fallahtafti, 2025). The optimized outputs from our algorithms can therefore be interpreted as assistance strategies that shape the hip extension moment profile in ways that may reduce muscular effort or enhance propulsion, consistent with observed human adaptation patterns in exoskeleton studies. Because our analysis relied on previously collected experimental data, we were constrained to these two parameters and could not include additional variables (e.g., onset timing, torque profile shape). We acknowledge this as a limitation, and future work should extend the framework to incorporate richer biomechanical descriptors for improved generalizability.

Regarding the augmented dataset, interpolation and controlled perturbations were used to expand the input space while preserving biomechanical plausibility, ensuring that torque profiles remained within safe and physiologically realistic ranges observed in prior exoskeleton trials (Slade et al., 2018; Slade et al., 2022; Gonabadi et al., 2020). This approach enhances robustness without introducing unrealistic patterns. Each ML model underwent hyperparameter tuning via grid-search or library optimization. For Gradient Boosting, tree depth, number of trees, and learning rate were tuned; for Random Forest, number of trees and depth; for SVR, kernel type and penalty factor; and for ridge regressions, regularization coefficients. Gaussian Process kernels were implemented with stable defaults. Overfitting risks, given the modest dataset size, were mitigated using repeated five-fold cross-validation (100 iterations). Although leave-one-subject-out validation would further address inter-individual variability, it was not implemented here and is acknowledged as future work.

For optimization algorithms, we included a diverse set spanning evolutionary, swarm-based, and Bayesian families to evaluate both exploration and exploitation strategies in a surrogate-based HIL context (Myunghee et al., 2017; Slade et al., 2018; Slade et al., 2022; Bryan et al., 2021; Ma et al., 2024; Echeveste and Bhounsule, 2025). Gradient-based methods were excluded because surrogate landscapes are highly nonlinear, often non-convex, and prone to local minima, making them less reliable for global exploration in this application (Slade et al., 2018; Slade et al., 2022). Finally, while this study focused on simulation-based evaluation, we recognize the ethical considerations in extending such optimization methods to clinical populations. Future patient trials will require careful Institutional Review Board (IRB) oversight, informed consent, and close monitoring to ensure participant safety, especially when testing algorithms that adaptively adjust torque in real time.

Ultimately, selecting the most appropriate optimization algorithm depends on the specific application goals and targeted population (Kutulakos and Slade, 2024). If the primary objective is to achieve the maximum possible reduction in metabolic cost, algorithms such as GSA may be preferred, even if they require more iterations or longer convergence times. However, prolonged walking trials may be impractical or fatiguing in clinical populations, such as individuals with mobility impairments. In these cases, faster-converging algorithms like PSO or CE, which can identify near-optimal solutions within fewer iterations, may be more suitable. For healthy individuals, tolerating longer optimization procedures to achieve greater metabolic reductions may be acceptable. This trade-off between convergence speed and optimization accuracy highlights the need for thoughtful algorithm selection tailored to the device context and the user population (Kutulakos and Slade, 2024).

Similar simulation-first approaches have been applied in other engineering domains, where surrogate modeling and optimization have effectively reduced experimental costs and accelerated iteration cycles. For example, sustainable additive manufacturing has leveraged computational optimization to minimize resource usage and emissions (Oladunni et al., 2025), while deep stable learning methods have enhanced predictive robustness under imbalanced data conditions (Xu et al., 2025). Drawing from these parallels, our framework highlights the potential of surrogate-informed HIL optimization to minimize costly real-world trial-and-error, thereby improving scalability and translational readiness.

Integrating biomechanics and motor adaptation perspectives further underscores the potential of surrogate-based frameworks to enhance exoskeleton control. Neuromuscular adaptation during exoskeleton use reflects gait plasticity, whereby users adjust stride patterns and muscle recruitment in response to assistance, often achieving improved energy efficiency over repeated sessions (Baud et al., 2021; Rodríguez-Fernández et al., 2021; Sanjeevi et al., 2021). Biofeedback mechanisms and perturbation-based approaches have been shown to accelerate adaptation, enabling users to refine coordination and reduce metabolic cost more rapidly (Desplenter and Trejos, 2018; Dzewaltowski et al., 2024; Lakmazaheri and Collins, 2025). Hierarchical control strategies, combining high-level intent recognition with torque modulation, account for such adaptive processes by incorporating within-stride variability into optimization (Slade et al., 2022; Echeveste and Bhounsule, 2025). In this context, the ability of the GB surrogate model to capture nonlinear parameter interactions parallels these adaptive responses, highlighting translational potential for tailoring assistance strategies to inter-individual adaptation dynamics.

In practical applications, surrogate models trained on data from healthy individuals could provide initial estimates of optimal assistance parameters for new users. These predictions could then be refined using limited personalized trials, enabling efficient customization of hip exoskeleton settings for clinical or real-world deployment. This simulation-based framework provides a foundation for translational application by identifying which optimization algorithms are most promising for HIL trials. In practice, surrogate-based insights can narrow the search space and initialize parameter settings, reducing the number of physical iterations required. The next step is to validate these algorithms in experimental HIL studies with the same hip exoskeleton device, where real-time metabolic feedback, user-specific adaptation, and fatigue effects can be directly assessed. Ultimately, this approach enables a more efficient pathway from simulation to clinical deployment, where optimization strategies can be tailored to patients and workers in rehabilitation and occupational settings.

### 4.3 Limitations

Although the surrogate-based optimization methods demonstrated promising performance, several limitations should be acknowledged. First, based on simulations run on a standard laptop, the reported computation times for each optimization algorithm may not directly translate to realistic experimental scenarios (Kutulakos and Slade, 2024). In human trials, optimization time is largely constrained not by computational latency but by the participant’s physiological response to torque perturbations and the duration required to reach a steady metabolic state (Mohammadzadeh Gonabadi et al., 2024b). Therefore, the purpose of reporting timing metrics in this study was to enable a controlled, relative comparison across algorithms under identical computational conditions, rather than to suggest real-world applicability of absolute convergence durations (Mohammadzadeh Gonabadi et al., 2024c). Second, the current simulations assume instantaneous feedback from the surrogate model, significantly accelerating the optimization process. In contrast (Mohammadzadeh Gonabadi et al., 2024c), real HIL experiments require waiting for the human body’s metabolic response to stabilize, often over several minutes per condition (Kutulakos and Slade, 2024). Thus, although BO or EBO required approximately 24–26 s to converge in the framework, actual implementation with human participants could take substantially longer due to biological delays and fatigue constraints. While the surrogate model captures key input–output relationships, its predictions do not account for biological variability, adaptation, or real-world gait dynamics. Thus, the reported 53% reduction should be interpreted as a model-based prediction, pending empirical validation in human-in-the-loop optimization experiments.

It is important to clarify that the reported runtimes (e.g., BO: 26 s) represent computational latency only and do not reflect the dominant biological stabilization periods required in real HIL experiments, which often extend to several minutes per condition and can accumulate to ∼2 h for a full optimization session. While the proposed surrogate-based framework offers substantial potential to reduce this experimental burden, its real-world speed remains constrained by participant fatigue and adaptation. As a limitation, future work should explicitly validate the true time savings in live HIL optimization experiments with the same exoskeleton system to confirm translational feasibility. While uncertainty was partially quantified through Gaussian Process confidence intervals, ensemble variance, and cross-validation variability, we acknowledge that real-world variability—particularly in clinical populations—may exceed model-based estimates. Future work should expand uncertainty quantification to better capture patient-specific unpredictability. While the present framework provides simulation-based predictions of optimal assistance patterns, these results have not yet been validated in real human-in-the-loop trials. Real-world experiments will be necessary to confirm the predicted metabolic cost reductions, capture adaptation and fatigue effects, and ensure clinical and translational relevance. The addition of error bars in Figure 6 helps convey variability in simulated outcomes, but physical trials remain the definitive step to establish robustness and generalizability. Our augmentation strategy perturbed input parameters to enrich surrogate training, which assumes that trial-to-trial variability in assistance settings can be represented as Gaussian noise. While this improved robustness, it does not directly model physiological variability or device measurement error, and future studies should examine whether input perturbations adequately reflect real-world variability in exoskeleton assistance.

Since our analysis relied on previously collected experimental data (Gonabadi et al., 2020; Antonellis et al., 2022; Dzewaltowski et al., 2024; Mohammadzadeh Gonabadi et al., 2024a), we were constrained to the torque parameters of Peak Magnitude and End Timing, and could not evaluate additional variables such as onset timing or torque profile shape. While these two parameters capture key biomechanical influences on hip assistance, this restricted scope limits generalizability. Future work should extend the framework to incorporate richer biomechanical descriptors and multi-parameter profiles to better reflect physiological variability and improve translational relevance. Furthermore, optimization metrics such as AUC and predicted metabolic cost were not directly linked to biomechanics outcomes (e.g., gait stability, neuromuscular adaptation), as the primary aim was to benchmark optimization algorithms for surrogate-based HIL personalization. This is acknowledged as a limitation, with future extensions aiming to integrate biomechanics-outcome analyses. Finally, while simulation provided valuable insights, extending these methods to clinical populations will require careful ethical oversight, informed consent, and participant monitoring in future patient trials.

The surrogate model was trained on pooled data from healthy adults, which may not fully capture inter-individual variability or the unique metabolic landscapes of clinical populations. Gait variability influenced by factors such as pathology, age, or fitness may amplify errors in optimization, as clinical users often adapt differently or require extended habituation (Antonellis et al., 2018; Leibman and Choi, 2025; Mohammadzadeh Gonabadi and Fallahtafti, 2025). Long-term neuromuscular plasticity and learning effects, while critical to sustained performance, were not captured in our simulations. Performance differences between healthy and clinical groups remain a key concern; individuals with spinal cord injury or post-stroke hemiparesis may experience greater gait instability or reduced adaptability, requiring population-specific retraining and experimental validation (Young and Ferris, 2017; Zhu et al., 2021; Edwards et al., 2022; Sado et al., 2022; Lee et al., 2025). Future studies should integrate adaptive learning models and validate across diverse cohorts to enhance translational potential.

Furthermore, this study’s optimization results were derived using a limited dataset from 10 healthy adults. While this cohort offers a proof-of-concept for healthy populations, it restricts the generalizability of the findings to broader user groups such as older adults, clinical populations, or individuals with gait impairments (Kutulakos and Slade, 2024). Expanding the dataset and incorporating more heterogeneous subject profiles would strengthen the robustness of the surrogate model and its predictive performance (Mohammadzadeh Gonabadi et al., 2024b). Another key limitation lies in the torque control strategy. Using static, piecewise semi-linear torque profiles, defined by Peak Magnitude and End Timing, simplifies the complexity of dynamic human gait. While effective for initial modeling, real-world gait is highly adaptive and phase-varying (Kutulakos and Slade, 2024). Future work should incorporate adaptive or time-varying torque profiles that respond to real-time biomechanical states. Additionally, the current framework (Kutulakos and Slade, 2024) does not incorporate physiological signals such as electromyography (EMG) (Lin et al., 2025), which could enhance surrogate modeling by capturing muscle activation patterns associated with metabolic demand. Future work could also explore incorporating other physiological or biomechanical outcomes, such as gait stability (Fallahtafti et al., 2021; Mohammadzadeh Gonabadi and Fallahtafti, 2025) or joint loading, into the simulation framework. Prior studies involving ankle exoskeletons have demonstrated that integrating EMG can refine assistance strategies and better align them with natural neuromuscular control (Lin et al., 2025). Extending this approach to hip exoskeletons could further personalize assistance by tailoring torque delivery to individual muscle responses. Future research should also work toward expanding the surrogate modeling framework to support time-varying, high-dimensional optimization scenarios (Kutulakos and Slade, 2024), leveraging real-time biomechanical and physiological inputs such as EMG to enhance adaptive control strategies across a wider range of users. Finally, the torque profile was modeled as a piecewise semi-linear function, which was selected based on the literature for computational efficiency and to approximate hip assistance torque in experimental conditions (Kutulakos and Slade, 2024; Mohammadzadeh Gonabadi et al., 2024a). While this approach is widely used, it oversimplifies the complexity of real gait dynamics and may reduce generalizability in clinical populations with irregular gait. Future work should explore more adaptive and physiologically informed profiles, such as EMG-driven or kinematic-based representations, to improve robustness and translational applicability.

## 5 Conclusion

This study introduced a surrogate-based HIL optimization framework for personalizing hip exoskeleton assistance. Among the evaluated ML regressors, GB demonstrated the highest predictive accuracy (RAEP = 0.66%). When paired with global optimizers, GSA predicted the lowest normalized metabolic cost (−1.06), while PSO and EBO exhibited superior convergence efficiency based on AUC and ARI metrics. Beyond individual algorithm performance, this framework approach offers a generalized methodology for screening optimization algorithms and hyperparameters before any human testing. Researchers developing new exoskeletons or assistive devices can gather a small set of assistance trials, fit a surrogate model, and run virtual HIL optimizations to identify the algorithms most likely to succeed in practice. In the clinic, the same surrogate can guide a brief calibration session in which the optimizer samples a few gait cycles and then recommends patient-specific peak-torque magnitudes and timing parameters, delivering a custom assistance profile in minutes rather than hours of metabolic titration. This process can significantly reduce experimental burden, enhance reproducibility, and streamline the deployment of personalized assistive strategies. Looking forward, this framework could be embedded into real-time adaptive controllers, enabling assistance profiles to update dynamically as patients adapt or as clinical needs evolve. Future research should validate these simulation-informed strategies in both healthy and clinical populations to ensure safety and translational viability. Ultimately, these steps will accelerate the deployment of exoskeleton technologies across rehabilitation, occupational, and performance domains, ensuring that optimization strategies remain responsive to diverse user populations. Beyond exoskeleton research, the methodological principles of surrogate-based optimization can be extended to other biomechanical and engineering applications where system-level sustainability and efficiency are critical. Similar strategies have been successfully applied in domains such as sustainable additive manufacturing (Oladunni et al., 2025), underscoring the transferability of these approaches to wearable robotics and supporting their potential for broader cross-disciplinary impact. Future work should validate surrogate-informed settings in human trials, incorporate physiological signals such as EMG, and extend the framework to time-varying, higher-dimensional, and adaptive controllers that can serve diverse users in real-world conditions.

## Data Availability

The original contributions presented in the study are included in the article/supplementary material, further inquiries can be directed to the corresponding author.
